# Effective public-private partnerships for sustainable antiretroviral therapy: outcomes of the Right to Care health services GP down-referral program

**DOI:** 10.1186/s12889-019-7660-x

**Published:** 2019-11-07

**Authors:** Idah Mokhele, Sello Mashamaite, Pappie Majuba, Thembi Xulu, Lawrence Long, Dorina Onoya

**Affiliations:** 1 Health Economics and Epidemiology Research Office, Department of Internal Medicine, School of Clinical Medicine, Faculty of Health Sciences, University of the Witwatersrand, Johannesburg, South Africa; 2 Right to Care, Johannesburg, South Africa; 3 Department of Global Health, Boston University School of Public Health, Boston, MA USA

**Keywords:** South Africa, Antiretroviral therapy, Loss to follow-up, Mortality, Viral load suppression, Private general practitioners, Down-referral

## Abstract

**Background:**

The recently increased access to antiretroviral therapy (ART) in South Africa has placed additional strain on human and infrastructure resources of the public health sector. Capacity from private-sector General Practitioners (GPs) could be leveraged to ease the current burden on the public health sector.

**Methods:**

We conducted a retrospective record review of routine electronic medical record data on a systematic sample of HIV-infected adults (≥18 years old) initiated on ART at a tertiary hospital outpatient HIV clinic in Johannesburg, South Africa and down-referred to private-GPs for continued care after stabilization on ART. We compared these patients (“GP down-referred”) to a control-cohort who remained at the referring site (“Clinic A”) and patients from a regional hospital outpatient HIV clinic not offering down-referral to GPs (“Clinic B”). Study outcomes assessed are viral load suppression (VL < 50 copies/ml) and attrition from care (all-cause-mortality or > 90-days late for a last-scheduled visit) by 12 months of follow-up following down-referral or eligibility.

**Results:**

A total of 3685 patients, comprising 373 (10.1%) GP down-referred, 2599 (70.5%) clinic A controls, and 713 (19.4%) clinic B controls were included in the analysis. Overall, 1535 patients (53.3%) had a suppressed viral load. A higher portion of GP down-referred patients had a suppressed viral load compared to clinic A and B patients (65.7% vs 49.1% vs 58.9%). After adjusting for demographic and baseline clinical covariates, we found no difference in viral load suppression between GP down-referred and control patients (adjusted relative risk [aRR] for clinic A vs GP down-referred 1.0; 95% CI: 0.9–1.1), (aRR for clinic B vs GP down-referred 1.0; 95% CI: 0.9–1.2).

Clinic B controls experienced the highest attrition compared to GP down-referred and clinic A controls (33.2% vs 11.3% vs 5.9%) and had a higher risk of attrition compared to GP down-referred patients (adjusted hazard ratio [aHR] 4.2; 95% CI: 2.8–6.5), whereas clinic B controls had a lower risk of attrition (aHR 0.5; 95% CI: 0.3–0.7).

**Conclusions and recommendations:**

Our results show that private-GPs can contribute to caring for stabilized public sector HIV patients on life-long ART. However, they require special efforts to improve retention in care.

## Background

South Africa has made substantial investments in HIV prevention and treatment policies and programs resulting in over 4 million of the 7.9 million people living with HIV initiating antiretroviral therapy (ART) through the public health sector, 55% reduction in HIV related deaths from 2010 to 2018, compared to 33% globally, and vast improvements in life expectancy [1–3].

In 2015 South Africa adopted the UNAIDS 90–90-90 targets aiming to: diagnose 90% of all people living with HIV; enrol 90% of diagnosed patients on sustained ART; and ensure that 90% of patients on ART achieve viral suppression [4]. To achieve these targets, in September 2016, South Africa further expanded ART access to include all persons diagnosed with HIV regardless of baseline CD4 count [5]. This universal-test-and-treat (UTT) strategy aims to increase early ART initiation to reduce morbidity and mortality further, and hopefully also reduce HIV transmission rates [5]. Though the country has made commendable progress, significant steps are still needed to close the gap and meet the target of 90% diagnosed patients on ART by 2020 [6]. The increased access to ART from these scale-up efforts is likely to place additional strain on the already burdened public health sector.

Currently, shortages of trained health care workers in the public health sector of South Africa constrain the delivery of quality health care [7, 8]. Projections made in 2012 estimated that an additional 6000 nurses, 11,000 counsellors, and 800 doctors are required, at an additional annual salary cost of ZAR 2.6 billion (US$ 400 million) to make universal access to HIV treatment a reality [7]. Recently, the public has sector has increased its service decentralisation and task-shifting efforts to maximise the use of lower-level cadres to increase capacity in the national HIV treatment program [9]. This task-shifting includes nurse-initiated and managed ART, decanting (down-referring) stable patients to primary health care (PHC) service level, integrating health services at PHC level, and reducing the number of scheduled patient visits to once every 2–3 months for stable patients [9, 10]. These creative and credible approaches have contributed positively to the national HIV treatment program. There has been a dramatic increase in HIV testing since before the national HIV testing campaign in 2010 [11, 12] leading to 85% of people living with HIV knowing their status, and overall ART coverage increasing from 20.3% in 2010 to over 60% in 2017 [13, 14]. However, the ART program will require additional capacity to adequately manage the anticipated increases in patient volumes as previously ART ineligible patients begin to access HIV treatment. Therefore, there is an urgent need to leverage the private-sector providers and secure additional capacity to manage stable patients on ART in the public health care sector.

The transfer of HIV infected, public sector patients who are stable on ART to private-sector medical practitioners for routine follow-up and management could ease the current public health sector burden. This model of decanting has been implemented in varying formats locally and in countries with emergent or overstretched public-sector ART programs, with differing outcomes [15–18]. Right to Care Health Services (RtCHS), a non-profit organisation working in the public health sector, implemented the general practitioners (GPs) down-referral model in the Gauteng province from 2011 to 2017. This involved partnership with the Provincial Department of Health and local private GPs. Eligibility criteria for down-referral to GPs included being initiated on, and remaining on a standard first-line ART regimen according to the prevailing South African HIV treatment guidelines for at least 12 months at the referring site, a viral load of < 400 copies/ml, CD4 count of > 250 cells/μl, no comorbidities and not on treatment for tuberculosis (TB) at down-referral.

The RtCHS GP down-referral program presents an opportunity to evaluate the outcomes of this down-referral model further and compare these to a public sector clinic program.

Previous versions of this model of care have had mixed results with some results leading to questioning the quality of care provided by private health providers to public sector patients while others demonstrated the feasibility of achieving acceptable results at lower or equivalent cost [15, 16, 19]. A GP down-referral program implemented in 2005 in the North West province, South Africa which had stabilised ART patients down-referred to local GPs similar to the RtCHS GP down-referral program demonstrated the ability of GPs to maintain patient treatment outcomes effectively. The cohort’s 12-month median viral loads were similar to down-referral levels (not deteriorating), and they also showed improved CD4 count over time [15]. However, these results were without direct comparison with a similar public sector cohort. Poor treatment outcomes reported from sexually transmitted infection (STI) [18, 20, 21] and malaria management by private GPs [22] has raised concerns about private GPs’ ability to manage patients according to national care and treatment guidelines. Additionally, the public sector HIV program context has changed considerably ever since the start of the national HIV treatment program, with changes in HIV clinical management guidelines, development of safer and cheaper ARV medications, and changes in diagnostic and lab monitoring requirements and costs.

The current evidence considering the viability of down-referral of public sector HIV infected patient for management by private medical practitioners, stated above, needs to be expanded to the current guideline-setting and to include a direct comparison with a similar public sector cohort. Our study aims to compare the treatment outcomes of HIV-positive patients’ enrolled in the RtCHS GP down-referral strategy compared to eligible but not down-referred patients from the referring site and eligible patients from a comparison, external public outpatient HIV clinic within a regional hospital.

## Methods

### Program description

The GP down-referral program was anticipated to be able to manage around two thousand patients through its ten partner GPs sites. The GPs were required to have completed an HIV/AIDS management course, have a dispensing license, and facilities for the proper storage of medicines. The public health sector supplied medicines pre-packed, batched by area and delivered to the GP for dispensing to patients. The referring public sector clinic (clinic A) used an electronic patient management system called TherapyEdge-HIV™ [23]. Right to Care supported the implementation of the TherapyEdge-HIV^TM^ for clinical management. A team of database management staff oversaw the design, security and maintenance of the systems, while a team of trained data management staff assisted with data capturing and routine data cleaning. In addition to clinical management of patients, the TherapyEdge-HIV™ system was also used for program monitoring and evaluation as well as operational research projects undertaken at the clinic.

GPs enrolled in the program were expected to use the TherapyEdge-HIV^TM^ system to capture medical care information for down-referred patients in their care. The National Health Laboratory Service (NHLS), the national contracted provider, provided laboratory monitoring, free of charge to the patient. NHLS had a direct interface with the TherapyEdge-HIV™ system, and GPs could access results directly through the system.

Patient eligibility criteria for down-referral included being initiated on, and remaining on a standard first-line ART regimen according to the prevailing South African national HIV treatment guidelines for at least 12 months at the referring site, a viral load of < 400 copies/ml, CD4 count of > 250 cells/μl at the time of down-referral. Patients on TB treatment and those with comorbidities such as renal disease, liver diseases, cardiovascular disease, neoplastic diseases and neurological disorders as well as those pregnant were excluded.

Patients were offered the option of down-referral after eligibility was confirmed. Consenting patients could then choose their preferred GP from the list of enrolled GPs based on the GP practices’ proximity to patients’ homes or work. All HIV-care services in the GP down-referral program were offered free of charge to down-referred patients.

Down-referred patients were dispensed two months of ART and were scheduled for their first appointment at the GP practice after that. They would see the GP every two months and go back to the referring clinic annually to take blood for clinical monitoring. Patients were managed according to national HIV treatment guidelines. RtCHS nurse case managers, who received mentoring from a RtCHS medical officer, reviewed the medical care provided by the GPs to ensure adherence to clinical guidelines and supported the GPs. Patients were referred back to the referring clinic if their viral load became unsuppressed, they had abnormal safety blood results or developed clinical problems. These included severe toxicities, opportunistic infections, abnormal weight loss, pregnancy and other significant findings. We defined loss to follow-up (LTFU) as having missed a scheduled visit by more than 90 days. LTFU patients were automatically up-referred to the referring site where counsellors would make up to three attempts to make telephonic contact with patients and urge them to return to care. All correspondence with the patients were captured in TherapyEdge-HIV™.

If GPs chose to leave the GP down-referral program, their allocated patients from the program would be referred back to the referring clinic, and patients would be given the option of selecting another GP if they chose to remain in the down-referral program. Alternatively, they could decide to continue their care at the referring clinic or transfer to another public sector facility.

### Study design and population

We conducted a retrospective record review of electronic, routinely collected medical record data, on a systematic sample of HIV-infected adults (> 18 years old at ART initiation) initiated on ART between 1 April 2004 and 31 December 2014. The patients were enrolled as follows:

1. GP down-referred: Patients from clinic A who were enrolled in the down-referral program and decanted to participating GPs. The referring clinic (clinic A) is a comprehensive HIV and AIDS care management and treatment (CCMT) clinic housed in a tertiary hospital in Johannesburg, South Africa. It has one of the largest cohorts of HIV-infected patients in the country and has been down-referring patients to local public-sector primary healthcare clinics since 2009 as a strategy to decongest the site and increase capacity for initiating more patients on ART [24]. For the GP down-referral program, stable patients were down-referred to 10 local private GPs. In our study cohort, the first down-referred patients were down-referred in October 2011, and the last in December 2015.
2. Clinic A control patients: Patients from clinic A who meet the GP down-referral eligibility criteria but were not decanted.
3. Clinic B control patients: Patients from a second HIV clinic who meet the GP down-referral eligibility criteria but were not decanted. Clinic B is a RtCHS- supported HIV clinic nested within a level 2 hospital in Krugersdorp, South Africa. Clinic B was not part of the GP down referral program, and none of their patients were offered the option of down-referral to a GP practice.

Clinic A and B eligible control patients are patients who met the study inclusion criteria and were eligible to be down-referred to the GP down-referral program but were not down-referred. We assessed control patients down-referral eligibility prospectively from January 2011 to December 2015, to align the timing of eligibility assessment to the year the GP down-referral program started. We excluded patients because of missing ART start date, being under 18 years old at ART start, initiating ART outside the study period and not meeting GP program eligibility criteria.

### Analytical variables

Clinical data were collected from all study sites, captured on-site and stored in an electronic patient management system, TherapyEdge-HIV™. Patient information collected includes socio-demographic factors including sex, age, nationality, highest education completed, and employment status. Baseline clinical characteristics include CD4 cell count, viral load, haemoglobin (Hb, measured in g/dL), and body mass index (BMI). CD4 was categorised as 250–349.9, 350–499.9, and ≥ 500 cells/μl. Viral load was categorised as < 50, 50–199 and 200–399 copies/ml. BMI was categorised as underweight (BMI < 18.5), normal (BMI 18.5 to < 25), overweight (BMI 25 to < 30) and obese (BMI > 30) kg/m^2^. Baseline clinical data analysed were reported 3 months before and after down-referral or first eligibility for clinic A and B control patients.

We calculated time on ART before down-referral eligibility as the difference between date of down-referral eligibility and ART initiation date, and categorised as 1 to 2.9 years, 3 to 4.9 years, and 5 or more years. Guideline year of ART initiation was categorised as 2004–2010 for patients initiated on ART between 1 April 2004 and 31 March 2010, and 2011–2014 for patients commencing ART from 1 April 2010 to 31 December 2014. Patients were followed up for 12 months from the date of entry into the GP down-referral program, or earliest date down-referral eligibility was met between January 2011 to December 2015 for control patients using individual-level de-identified electronic medical record data. We created a dichotomous variable classified as ‘yes’ or ‘no’, to indicate if patients experienced comorbid conditions during the 12-month study follow-up period. Where ‘yes’ referred to any, and earliest report of any comorbid condition according to lab monitoring results or clinical report from the patient’s electronic file, and ‘no’ was defined as never having a comorbid condition reported during the study follow-up period.

The primary exposure variable was the being enrolled in the GP down-referral compared to clinic A and B control patients.

The primary study outcome is attrition from HIV care (all-cause mortality/ LTFU) during the 12 months observation period after down-referral or becoming eligible for down-referral (for control patients). The secondary outcome is viral load (VL) suppression to undetectable levels (VL < 50 copies/ml) [25, 26], viral loads analysed were reported six months before and after the end of the 12-month follow-up period.

### Statistical analysis

Baseline characteristics at down-referral or down-referral eligibility for control site patients were described using medians and interquartile ranges (IQRs) for continuous variables and proportions and corresponding percentages for categorical variables stratified by cohort (GP down-referred vs clinic A and B patients).

Modified Poisson regression with robust standard errors was used to estimate the relative risk (RR) of viral load suppression to undetectable levels (VL < 50 copies/ml), and the corresponding 95% confidence intervals (95% CIs). Factors identified with a univariate *p*-value < 0.1 and a priori variables of importance were included in the multivariate model. We report adjusted relative risk (aRR) and 95% CIs.

We used time to event analysis to compare attrition from care between the GP down-referral and control cohorts. Person-time accrued from the date of down-referral for patients enrolled in the GP down-referral program, and date of eligibility for down-referral for patients in the control cohorts. For the attrition outcome, patients were followed-up until the last date seen at the facility (for deceased/LTFU), or completion of the 12 months of follow-up. Cox proportional-hazard regression was used to identify predictors of attrition from care. Factors identified with a univariate *p*-value < 0.1 and a priori variables of importance in attrition from care in the crude hazards ratio (HR) were included in the multivariate models. We report adjusted hazards ratio (aHR) and 95% CIs. The assumption of proportionality of hazards was tested using Schoenfeld residuals.

Data analysis was conducted using STATA version 14 (StataCorp, College Station, TX).

## Results

### Selection of study sample

**Fig. 1:**
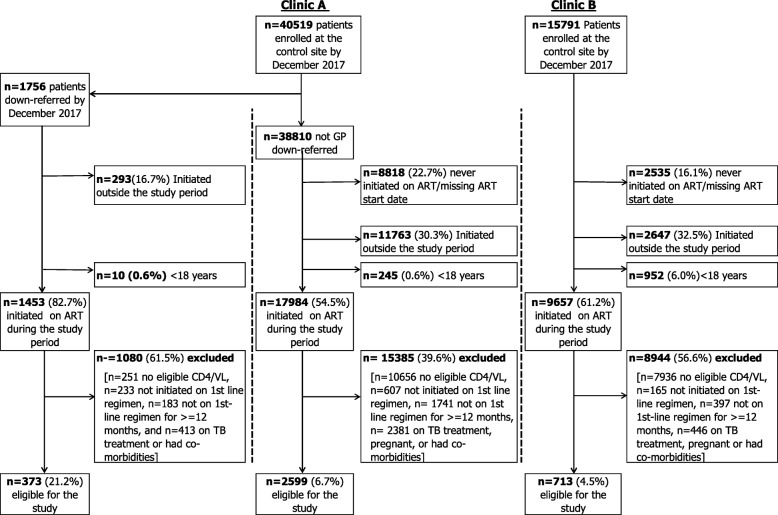
Selection of the study cohort, by study site

Figure 1 provides a summary of the screening process for participant inclusion into the study. A total of 3685 patients, consisting of 373 (10.1%) GP down-referred, 2599 (70.5%) clinic A, and 713 (19.4%) clinic B controls.

### Demographic characteristics

Table 1 presents the demographic and clinical characteristics of the study cohort at down-referral or down-referral eligibility. Overall, 66.2% were female, and the median age at down-referral or eligibility was 39.3 years (IQR 33.9–45.7). Over two-thirds of the study population (66.9%) had a secondary school education or completed grade 12, with clinic B controls having a higher proportion (60.7%) of patients completing grade 12 than GP down-referred (48.3%) and clinic A controls (42.6%). A much higher percentage of GP down-referred (63.8%) and clinic A controls (55.0%) were employed compared to clinic B controls (29.3%).

**Table 1:** Demographic and baseline clinical characteristics of the study sample (*n* = 3685)

	GP down-referred	Clinic A controls	Clinic B controls	Total
	373 (10.1)	2599 (70.5)	713 (19.4)	3685 (100.0)
	*n* (%)	*n* (%)	*n* (%)	*n* (%)
Sex				
Female	258 (69.2)	1717 (66.1)	463 (64.9)	2438 (66.2)
Male	115 (30.8)	882 (33.9)	250 (35.1)	1247 (33.8)
Age at down-referral/eligibility (years) Median (IQR)	40.6 (35.9–47.2)	38.9 (33.8–44.9)	39.7 (33.3–47.4)	39.3 (33.9–45.7)
18–29.99	21 (5.6)	301 (11.6)	109 (15.3)	431 (11.7)
30–39.99	150 (40.2)	1117 (43.0)	251 (35.2)	1518 (41.2)
40–49.99	133 (35.7)	855 (32.9)	224 (31.4)	1212 (32.9)
50+	69 (18.5)	326 (12.5)	129 (18.1)	524 (14.2)
Nationality				
South African	316 (84.7)	2281 (87.8)	696 (97.6)	3293 (89.4)
Non-South African	57 (15.3)	318 (12.2)	17 (2.4)	392 (10.6)
Education level				
Primary school or less	58 (15.5)	386 (14.9)	216 (30.3)	660 (17.9)
Some secondary school	105 (28.2)	685 (26.4)	28 (3.9)	818 (22.2)
> =Grade 12	180 (48.3)	1107 (42.6)	433 (60.7)	1720 (46.7)
Missing	30 (8.0)	421 (16.2)	36 (5.0)	487 (13.2)
Employment status				
Employed	238 (63.8)	1430 (55.0)	209 (29.3)	1877 (50.9)
Unemployed	130 (34.9)	1125 (43.3)	472 (66.2)	1727 (46.9)
Missing	5 (1.3)	44 (1.7)	32 (4.5)	81 (2.2)
Time on ART at down-referral/eligibility (years) Median (IQR)	4.2 (2.7–6.3)	3.0 (1.9–5.0)	2.1 (1.5–3.7)	3.0 (1.8–4.9)
1–2.9 year	104 (27.9)	1281 (49.3)	480 (67.3)	1865 (50.6)
3–4.9 years	111 (29.8)	678 (26.1)	136 (19.1)	925 (25.1)
> = 5 years	158 (42.4)	640 (24.6)	97 (13.6)	895 (24.3)
Guideline year of ART initiation				
2004–2010	205 (55.0)	1828 (70.3)	361 (50.6)	2394 (65.0)
2011–2014	168 (45.0)	771 (29.7)	352 (49.4)	1291 (35.0)
Baseline CD4 (cells/μl)				
250–349.9	152 (40.8)	679 (26.1)	196 (27.5)	1027 (27.9)
350–499.9	137 (36.7)	854 (32.9)	228 (32.0)	1219 (33.1)
> =500	84 (22.5)	1066 (41.0)	289 (40.5)	1439 (39.1)
Baseline VL (copies/ml)				
< 50	297 (79.6)	1466 (56.4)	475 (66.7)	2238 (60.8)
50–199	61 (16.4)	698 (26.9)	151 (21.2)	910 (24.7)
200–399	15 (4.0)	434 (16.7)	86 (12.1)	535 (14.5)
Baseline Haemoglobin (g/dL) Med (IQR)	14.0 (13.0–14.8)	13.9 (12.9–15.0)	14.0 (13.0–14.9)	13.9 (12.9–14.9)
Baseline BMI (kg/m^2^)				
Normal	241 (64.6)	993 (38.2)	291 (40.8)	1525 (41.4)
Overweight	82 (22.0)	593 (22.8)	158 (22.2)	833 (22.6)
Obese	46 (12.3)	350 (13.5)	101 (14.2)	497 (13.5)
Missing	4 (1.1)	663 (25.5)	163 (22.9)	830 (22.5)
ART regimen				
TDF + 3TC/FTC + EFV/NVP	172 (46.1)	754 (29.4)	342 (48.0)	1268 (34.8)
AZT + 3TC + EFV/NVP	18 (4.8)	152 (5.9)	24 (3.4)	194 (5.3)
d4T + 3TC + EFV/NVP	183 (49.1)	1656 (64.6)	347 (48.7)	2186 (59.9)
Comorbidities in the 12 month follow-up				
No	366 (98.1)	2529 (97.3)	694 (97.3)	3589 (97.4)
Yes	7 (1.9)	70 (2.7)	19 (2.7)	96 (2.6)
12 month Retention				
Alive and in care	331 (88.7)	1736 (66.8)	367 (51.5)	2434 (66.1)
LTFU	42 (11.3)	83 (3.2)	175 (24.5)	300 (8.1)
Deceased	0	26 (1.0)	7 (1.0)	33 (0.9)
Transferred out	0	754 (29.0)	164 (23.0)	918 (24.9)
12 month-VL suppression (< 50 copies/mL)				
Unsuppressed	119 (31.9)	967 (37.2)	260 (36.5)	1346 (36.5)
Suppressed	228 (61.1)	934 (35.9)	373 (52.3)	1535 (41.7)
Missing	26 (7.0)	698 (26.9)	80 (11.2)	804 (21.8)

IQR, interquartile range; ART, antiretroviral therapy; VL, viral load; BMI, body mass index; 3TC, Lamivudine; EFV, efavirenz; TDF, tenofovir; NVP, Nevirapine; AZT, Zidovudine; FTC, emtricitabine; d4T, stavudine

### Baseline clinical characteristics

Overall, 55.0% of GP down-referred and 50.6% clinic B controls were initiated on ART in the 2004–2010 national HIV guideline period compared to 70.3% of the clinic A controls. A total of 758 (20.6%) patients were initiated on ART between 2011 and 2014 after the GP down-referral program started and the majority of these patients (93.9%) were on ART for 1–2.99 years before down-referral eligibility. A total of 158 (42.4%) of GP down-referred patients were on treatment for five years or more before down-referral, compared to clinic A controls (24.6%) and clinic B controls (13.6%).

Over a third (36.1%) of total study cohort presented with a high body mass index (BMI), whereas a higher portion of GP down-referred patients presented with a normal BMI at down-referral compared to clinic A and B patients (64.6% vs 38.2% vs 40.8%). A higher proportion of clinic A controls (41.0%) and clinic B controls (40.5%) presented with CD4 counts > 500 cells/μl compared to GP down-referred patients (22.5%). Overall, 79.6% of the GP down-referred study cohort presented with a viral load < 50 copies/ml compared to almost 60% of the combined control cohort (clinic A and B). Most of clinic A controls (64.6%) were on Stavudine (d4T)-based first-line regimens at down-referral eligibility, compared to 49.1% GP down-referred patients and 48.7% of clinic B controls.

A total of 2.6% of the study cohort experienced comorbid conditions during the 12-month study follow up period. With a higher proportion of clinic A (2.7%) and clinic B controls (2.7%), patients experiencing comorbid conditions compared to 1.9% of GP down-referred patients. Of these, the majority experienced anaemia (45.8%) followed by renal dysfunction (31.2%), while 22.9% had signs of liver injury.

When we explored experiencing comorbid conditions further using time to event analysis (only reporting crudes), 96 patients experienced conditions during 2888.2 person-years of observation (incidence rate 3.3/100 person-years, 95% CI: 2.7–4.1) (Table 2). Patients who experienced all-cause mortality by the end of the study follow-up period were more likely to experience a comorbid condition (cHR for 2011–2014 vs 2004–2010 0.6; 95% CI: 0.4–0.9), and (cHR for deceased vs alive and in care 18.0; 95% CI: 6.3–50.6). While being initiated on a d4t-based ART regimen and being initiated in 2011–2014 guideline period was protective (cHR for d4T + 3TC + EFV/NVP vs TDF + 3TC/FTC + EFV/NVP 0.6; 95% CI: 0.4–0.9), (cHR for 2011–2014 vs 2004–2010 0.6; 95% CI: 0.4–0.9).

**Table 2:** Predictors of experiencing comorbid conditions during the 12 months follow-up after down-referral or eligibility

	Experienced comorbid conditions *n* (%)	Person-years	Rate per 100 person-years (95% CI)	cHR (95% CI)
	96 (2.6)	2888.2	3.3 (2.7–4.1)	
Study cohort				
GP down-referred	7 (1.9)	368.7	1.9 (0.9–4.0)	1
Clinic A controls	70 (2.7)	2011.8	3.5 (2.8–4.4)	2.0 (0.9–4.2)
Clinic B controls	19 (2.7)	507.6	3.7 (2.4–5.9)	2.2 (0.9–5.3)
Sex				
Female	70 (2.9)	1900.8	3.7 (2.9–4.7)	1
Male	26 (2.1)	987.4	2.6 (1.8–3.9)	0.7 (0.5–1.1)
Age at down-referral/eligibility (years)				
18–29.99	6 (1.4)	348.7	1.7 (0.8–3.8)	1
30–39.99	37 (2.4)	1195.4	3.1 (2.2–4.3)	1.8 (0.8–4.3)
40–49.99	38 (3.1)	940.5	4.0 (2.9–5.5)	2.4 (0.9–5.6)
50+	15 (2.9)	403.6	3.7 (2.2–6.2)	2.2 (0.8–5.6)
Nationality				
South African	82 (2.5)	2548.3	3.2 (2.6–4.0)	1
Non-South African	14 (3.6)	339.8	4.1 (2.4–7.0)	1.2 (0.7–2.2)
Education level				
Primary school or less	16 (2.4)	519.9	3.1 (0.9–5.0)	1.0 (0.6–1.8)
Some secondary school	30 (3.7)	711.2	4.2 (2.9–6.0)	1.3 (0.8–2.2)
> =Grade 12	41 (2.4)	1352.4	3.0 (2.2–4.1)	1
Employment status				
Employed	49 (2.6)	1509.0	3.2 (2.5–4.3)	1
Unemployed	42 (2.4)	1322.4	3.2 (2.3–4.3)	1.0 (0.7–1.5)
Time on ART at down-referral/eligibility (years)				
1–2.9 year	54 (2.9)	1526.8	3.5 (2.7–4.6)	1
3–4.9 years	18 (1.9)	713.0	2.5 (1.6–4.0)	0.7 (0.4–1.2)
> =5 years	24 (2.7)	648.3	3.7 (2.5–5.5)	1.1 (0.7–1.8)
Guideline year of ART initiation				
2004–2010	49 (2)	1832.5	2.7 (2.0–3.5)	1
2011–2014	47 (3.6)	1055.7	4.5 (3.3–5.9)	0.6 (0.4–0.9)
Baseline CD4 (cells/μl)				
250–349.9	30 (2.9)	852.9	3.5 (2.5–5.0)	1.3 (0.8–2.2)
350–499.9	38 (3.1)	968.1	3.9 (2.9–5.4)	1.5 (0.9–2.4)
> =500	28 (1.9)	1067.1	2.6 (1.8–3.8)	1
Baseline VL (copies/mL)				
< 50	61 (2.7)	1750.7	3.5 (2.7–4.5)	1
50–199	23 (2.5)	715.3	3.2 (2.1–4.8)	0.9 (0.6–1.5)
200–399	11 (2.1)	421.1	2.6 (1.4–4.7)	0.8 (0.4–1.4)
Baseline BMI (kg/m^2^)				
Normal	46 (3)	1297.0	3.5 (2.7–4.7)	1
Overweight	20 (2.4)	706.6	2.8 (1.8–4.4)	0.8 (0.5–1.4)
Obese	17 (3.4)	411	4.1 (2.6–6.7)	1.2 (0.7–2.0)
ART regimen				
TDF + 3TC/FTC + EFV/NVP	47 (3.7)	1036.9	4.5 (3.4–6.0)	1
AZT + 3TC + EFV/NVP	4 (2.1)	151.6	2.6 (1.0–7.0)	0.6 (0.2–1.6)
d4T + 3TC + EFV/NVP	43 (2)	1666.5	2.6 (1.9–3.5)	0.6 (0.4–0.9)
12 month Retention				
Alive and in care	81 (3.3)	2300.3	3.5 (2.8–4.4)	1
LTFU	5 (1.7)	175.3	2.9 (1.2–6.7)	1.1 (0.5–2.5)
Deceased	4 (12.1)	10.2	39.3 (14.7)	18.0 (6.3–50.6)
Transferred out	6 (0.6)	401.4	1.5 (0.7–3.3)	0.5 (0.2–1.3)

HR, hazard ratio, cHR, crude hazard ratio, 95% CI, 95% confidence interval, PY, person-years

### Predictors of viral suppression at 12 months post-down-referral or first eligibility

By the end of the 12 month study follow-up period, 2881 of the 3685 (78.2%) total study cohort had a 12-month viral load available. Clinic A control patients had the highest proportion (26.9%) of patients missing a 12-month viral load compared to GP down-referred (7.0%) and clinic B controls (11.2%). Those with missing 12-month viral loads were similar to those with 12-month viral loads in terms of demographic factors, except for sex and employment. A higher proportion was male, employed, initiated on ART during the 2004–2010 guideline period. A higher percentage was also on treatment for three years or longer at down-referral, had a baseline CD4 counts of < 500, were initiated on a d4t based ARV regimen and were transferred out (Additional file 1).

Of the patients that had a 12-month viral load result, 1535/2881 (53.3%) had a suppressed viral load at 12 months (Table 3). A higher portion of GP down-referred patients had a suppressed 12-month viral load compared to clinic A and B patients (65.7% vs 49.1% vs 58.9%). After adjusting for demographic and baseline clinical baseline covariates, there was no difference in 12-month viral load suppression between GP down-referred and control patients (aRR for clinic A vs GP down-referred 1.0; 95% CI: 0.9–1.1), (aRR for clinic B vs GP down-referred 1.0; 95% CI: 0.9–1.2). Those with a viral load of 50–399 copies/ml at down-referral or eligibility were less likely to achieve viral load suppression at 12 months compared to those that had a viral load of < 50 copies/ml (aRR for 50–149 vs < 50 0.1; 95% CI: 0.1–0.2, aRR for 150–299 vs < 50 0.1; 95% CI: 0.1–0.1, aRR for 300–399 vs < 50 0.04; 95% CI: 0.04–0.1). Compared to patients alive and in care at 12-months, LTFU patients were less likely to achieve viral load suppression (aRR 0.8; 95% CI: 0.7–0.9).

**Table 3:** Predictors of suppressed viral load (< 50 copies/mL) at 12-month post-down-referral/ eligibility

	Suppressed VL (1535/2881)	RR	aRR
	*n* (%)	(95% CI)	(95% CI)
Study cohort			
GP down-referred	228 (65.7)	1	1
Clinic A controls	934 (49.1)	0.7 (0.7–0.8)	1.0 (0.9–1.1)
Clinic B controls	373 (58.9)	0.9 (0.8–0.99)	1.0 (0.9–1.2)
Sex			
Female	1036 (53.7)	1	1
Male	499 (52.5)	1.0 (0.9–1.1)	1.0 (0.9–1.0)
Age at down-referral/eligibility (years) Median (IQR)			
18–29.99	193 (55.3)	1	1
30–39.99	657 (55.8)	1.0 (0.9–1.1)	1.0 (0.9–1.1)
40–49.99	481 (51.4)	0.9 (0.8–1.0)	0.9 (0.9–1.0)
50+	204 (48.8)	0.9 (0.8–1.0)	1.0 (0.9–1.1)
Nationality			
South African	1363 (53.2)	1	
Non-South African	172 (53.9)	1.0 (0.9–1.1)	
Education level			
Primary school or less	275 (51.7)	1.0 (0.9–1.0)	
Some secondary school	350 (53.9)	1.0 (0.9–1.1)	
> =Grade 12	765 (54.2)	1	
Employment status			
Employed	768 (53.6)	1	
Unemployed	731 (52.9)	1.0 (0.9–1.1)	
Time on ART at down-referral/eligibility (years) Median (IQR)			
1–2 year	860 (54.6)	1	1
3–4 years	350 (51.8)	0.9 (0.9–1.0)	1.0 (0.9–1.1)
> =5 years	325 (51.7)	0.9 (0.9–1.0)	0.9 (0.9–1.0)
Guideline year of ART initiation			
2004–2010	929 (52.2)	1	
2010–2014	606 (54.9)	1.1 (0.98–1.1)	
Baseline CD4 (cells/μl)			
250–349.9	472 (54.5)	1.0 (0.96–1.0)	
350–499.9	510 (53.6)	1.0 (0.9–1.1)	
> =500	553 (52.0)		
Baseline Haemoglobin (g/dL)	**–**	1.0 (0.97–1.0)	
Baseline VL (copies/mL)			
< 50	1431 (81.4)	1	1
50–149	61 (10.9)	0.1 (0.1–0.2)	0.1 (0.1–0.2)
150–299	33 (8.1)	0.1 (0.1–0.1)	0.1 (0.1–0.1)
300–399	10 (6.5)	0.1 (0.04–0.1)	0.1 (0.04–0.1)
Baseline BMI (kg/m2)			
Normal	715 (54.1)	1	
Overweight	382 (52.4)	1.0 (0.9–1.1)	
Obese	222 (52.6)	1.0 (0.9–1.1)	
ART regimen			
TDF + 3TC/FTC + EFV/NVP	595 (55.7)	1	1
AZT + 3TC + EFV/NVP	100 (60.2)	1.1 (0.9–1.2)	1.0 (0.9–1.1)
d4T + 3TC + EFV/NVP	825 (51.0)	0.9 (0.9–0.98)	1.0 (0.9–1.0)
12 month Retention			
Alive and in care	1121 (55.5)	1	1
LTFU	117 (47.0)	0.9 (0.8–0.99)	0.8 (0.7–0.9)
Deceased	16 (55.2)	1.0 (0.7–1.4)	1.0 (0.8–1.2)
Transferred out	281 (51.6)	0.9 (0.9–1.0)	0.9 (0.9–1.0)

RR, risk ratio; aRR, adjusted relative risk; 95% CI, 95% confidence interval

### Predictors of attrition from care at 12-month post-down-referral

Table 4 provides rates and predictors of attrition from care for the study cohort resulting from mortality or LTFU. Overall, 333 (12.0%) patients were deceased or LTFU in the 2480.2 person-years of observation (incidence rate 13.4/100 person-years, 95% CI: 12.1–14.9). Clinic B controls experienced the highest attrition (33.2%) during the 12 month follow-up period (incidence rate 41.9/100 person-years, 95% CI: 36.2–48.4) compared to 11.3% of GP down-referred patients (incidence rate 11.5/100 person-years, 95% CI: 8.5–15.6) and 5.9% among the clinic A controls (incidence rate 6.5/100 person-years, 95% CI: 5.4–7.8).

**Table 4:** Predictors of attrition from care after 12 months follow-up after down-referral

	Deceased / LTFUn (%)	Person-years	Rate per 100 person-years (95% CI)	HR (95% CI)	aHR (95% CI)
Study cohort	333 (12.0)	2480.2	13.4 (12.1–14.9)		
GP down-referred	42 (11.3)	364.5	11.5 (8.5–15.6)	1	1
Clinic A controls	109 (5.9)	1681.1	6.5 (5.4–7.8)	0.6 (0.4–0.8)	0.5 (0.3–0.7)
Clinic B controls	182 (33.2)	434.5	41.9 (36.2–48.4)	3.6 (2.6–5.1)	4.2 (2.8–6.5)
Sex					
Female	213 (11.7)	1627.9	13.1 (11.4–15.0)	1	
Male	120 (12.6)	852.3	14.1 (11.8–16.8)	1.1 (0.9–1.3)	
Age at down-referral/eligibility (years)					
18–29.99	39 (11.7)	302.0	12.9 (9.4–17.6)	1	
30–39.99	118 (10.3)	1032.0	11.4 (9.5–13.7)	0.9 (0.6–1.3)	
40–49.99	118 (13.1)	801.0	14.7 (12.3–17.6)	1.1 (0.8–1.6)	
50+	58 (14.9)	344.5	16.8 (13.0–21.8)	1.3 (0.9–1.9)	
Nationality					
South African	323 (13.3)	2167.9	14.9 (13.4–16.6)	1	1
Non-South African	10 (3.0)	312.3	3.2 (1.7–6.0)	0.2 (0.1–0.4)	0.2 (0.1–0.6)
Education level					
Primary school or less	70 (13.9)	450.6	15.5 (12.3–19.6)	1.0 (0.8–1.3)	1.0 (0.7–1.4)
Some secondary school	60 (8.3)	665.8	9.0 (7.0–11.6)	0.6 (0.4–0.8)	1.1 (0.7–1.6)
> =Grade 12	179 (13.5)	1173.2	15.3 (13.2–17.7)	1	1
Employment status					
Employed	143 (9.9)	1314.7	10.9 (9.2–12.8)	1	1
Unemployed	174 (13.7)	1117.3	15.6 (13.4–18.1)	1.4 (1.1–1.8)	1.0 (0.7–1.3)
Time on ART at down-referral/eligibility (years)					
1–2.99 year	202 (12.9)	1386.0	14.6 (12.7–16.7)	1	1
3–4.99 years	58 (9.1)	589.1	9.8 (7.6–12.7)	0.7 (0.5–0.9)	1.1 (0.7–1.7)
> =5 years	73 (12.9)	505.1	14.5 (11.5–18.2)	1.0 (0.8–1.3)	1.9 (1.2–3.0)
Guideline year of ART initiation					
2004–2010	156 (9.5)	1495.5	10.4 (8.9–12.2)	1	1
2010–2014	177 (15.7)	984.6	18.0 (15.5–20.8)	1.7 (1.4–2.1)	1.1 (0.5–1.6)
Baseline CD4 (cells/μl)					
250–349.9	90 (10.6)	774.5	11.6 (9.5–14.3)	0.8 (0.6–0.99)	0.7 (0.5–1.0)
350–499.9	111 (11.8)	842.4	13.2 (10.9–15.9)	0.9 (0.7–1.1)	0.8 (0.6–1.1)
> =500	132 (13.4)	863.3	15.3 (12.9–18.1)	1	1
Baseline Haemoglobin (g/dL)	**–**	1816.6	12.1 (10.6–13.8)	0.9 (0.8–1.0)	
Baseline VL (copies/mL)					
< 50	218 (13.0)	1499.1	14.5 (12.7–16.6)	1	1
50–149	51(9.4)	489.1	10.4 (7.9–13.7)	0.7 (0.5–0.97)	0.8 (0.6–1.2)
150–299	43 (10.9)	355.1	12.1 (9.0–16.3)	0.8 (0.6–1.2)	1.3 (0.9–1.8)
300–399	20 (13.3)	135.5	14.8 (9.5–22.9)	1.0 (0.6–1.6)	1.5 (0.9–2.7)
Baseline BMI (kg/m2)					
Normal	142 (10.7)	1213.5	11.7 (9.9–13.8)	1	1
Overweight	57 (8.1)	654.2	8.7 (6.7–11.3)	0.7 (0.5–1.0)	0.7 (0.5–0.9)
Obese	50 (11.7)	385.2	13.0 (9.8–17.0)	1.1 (0.8–1.5)	1.0 (0.7–1.4)
ART regimen					
TDF + 3TC/FTC + EFV/NVP	185 (16.7)	967.1	19.1 (16.6–22.1)	1	1
AZT + 3TC + EFV/NVP	11 (7.8)	126.5	8.7 (4.8–15.7)	0.5 (0.2–0.8)	0.5 (0.2–1.1)
d4T + 3TC + EFV/NVP	136 (9.2)	1355.3	10.0 (8.5–11.9)	0.5 (0.4–0.7)	0.4 (0.2–0.7)

HR, hazard ratio, aHR, adjusted hazard ratio, 95% CI, 95% confidence interval, PY, person-years

In multivariable analysis, after adjusting for baseline factors, clinic B controls had over four times the risk of attrition from care compared to GP down-referred patients (aHR for clinic B vs GP down-referred 4.2; 95% CI: 2.8–6.5). Clinic A controls were 50% less likely to be deceased or LTFU compared to GP down-referred patients (aHR for clinic A vs GP down-referred 0.5; 95% CI: 0.3–0.7). Compared to South Africans, Non-South African patients were less likely to be deceased or LTFU (0.2; 95% CI: 0.1–0.6), as well as those overweight at down-referral (aHR for overweight BMI vs normal BMI 0.7; 95% CI: 0.5–0.9), and those that were initiated on d4t-based ART regimens (aRR for d4T + 3TC + EFV/NVP vs TDF + 3TC/FTC + EFV/NVP 0.4; 95% CI: 0.2–0.7). Longer time on ART before down-referral eligibility led to almost double the risk of attrition from care (aHR for > = 5 years vs 1–2.99 years 1.9; 95% CI: 1.2–3.0).

## Discussion

We compared treatment outcomes between patients down-referred to private GPs and public sector patients after becoming stable on ART. We defined treatment outcomes as viral load suppression to undetectable levels (< 50 copies/ml), and attrition from care based on all-cause mortality or having no contact with the treatment site for 90 days or more after missing the last scheduled visit during the 12 month study follow-up period after down-referral or down-referral eligibility for control site patients. Our results show that down-referred public sector patients have similar virological outcomes compared to public sector control patients. However, down-referred patients had poorer retention outcomes.

RtCHS provided programmatic and clinical oversight to the GPs; strengthening their capacity to provide HIV care according to national HIV treatment guidelines. Only 7% of the GP-down-referred had missing 12-month viral loads, demonstrating GPs’ high adherence to viral load monitoring required, as stated in the national HIV treatment guidelines [27], which is much lower than missing rates reported in the public health sector in South Africa, where missing rates of 31% were recently reported [28]. Moreover, although a higher proportion of GP managed patients had a suppressed viral load than clinic A and clinic B controls, there was no statistical difference in achieving viral load suppression to undetectable levels (< 50 copies/ml) between the study cohorts.

Our results are supportive of results from a donor-funded, private GP-run outpatient clinic in rural Mpumalanga, in South Africa initiating treatment naïve patient on ART that also demonstrated good treatment outcomes. They reported higher rates of viral suppression (70%) [29] and the cohort’s 12-month virological outcomes were comparable to public sector programs at the time [29, 30]. The GP down-referral program used a viral load of < 400 copies/ml as one of the eligibility criteria specifying stabilization in addition to immunological (CD4 > 250 cells/μl) and clinical (no comorbidities) criteria. However, CD4 count (immunological stability) at down-referral eligibility was not a significant predictor of 12-month virologic suppression or retention in this study cohort. Additionally, very few patients (2.6%) experienced a comorbid condition during the 12-month follow-up period. Reported comorbidities might be underestimated because of attrition as comorbid conditions have been reported as barriers to retention, and may go unreported among those LTFU and deceased [31].

Patients presenting with viral loads of 50–399 copies/ml were less likely to have a suppressed viral load at 12-month follow-up compared to those presenting with viral loads of < 50 copies/ml. Although the national HIV treatment program considers patients with a viral load of < 400 copies/ml stabilised on treatment, the viral load algorithm from the HIV treatment guidelines recommend intensified adherence support and also stresses the need for compliance to viral monitoring requirements for these patients. Future down-referral programs to private sector providers may need to strengthen providers’ routine adherence support to patients with viral loads above 50–400 copies/ml as indicated in the adherence guidelines for HIV TB and non-communicable diseases, to ensure improved treatment outcomes as stated in the adherence guidelines for HIV TB and non-communicable diceases [32].

Results from a study comparing patients from the GP down-referral program’s referring site with patients from a private GP managed outpatient clinic in Johannesburg also found a higher proportion of GP managed patients with suppressed viral loads at 12-month follow up [33]. The same study also found higher LTFU rates among private GP managed patients with that risk of LTFU increasing over time [33]. In our cohort, clinic B controls had higher rates of attrition from care and were four times at risk compared to GP down-referred patients. However, clinic A controls had a 50% less risk of attrition compared to GP down-referred patients. The referring site makes rigorous efforts to follow-up on patients before confirming LTFU. Although the GP down-referred patients may also benefit from these efforts besides the follow-up by the RtCHS call centre, it seems it is not enough to reduce attrition from care. The follow up by the referring site may only happen long after the initial missed visits when patients are up-referred. The convenience provided in GP managed care may introduce some unforeseen challenges to retention. GPs care for down-referred patients is limited to HIV care services, anything beyond this such as injuries or infections developing after down-referral would require the patient to go back to the referring site to be able to access these additional services free of charge. Patients may choose to access these additional services from the GPs, at their own cost, rather than going through the inconvenience of returning to the referring site. These costs may escalate beyond what the patient can afford and ultimately risks patients disengaging from care [34]. LTFU may be self-transfers to other health facilities or unreported mortality [35]. In the GP down-referral program transferring out to another site requires up-referral to the referring site, which may deter patients from following proper procedure for transferring out of the site resulting in self-transfer. If the procedure for patient transfers could be more streamlined, leveraging the electronic data management system used in the program, it could reduce the risk of undocumented self-transfers.

Longer time on ART before down-referral eligibility led to almost double the risk of attrition from care. Experienced patients may no longer be as motivated to remain in care because of treatment fatigue as a consequence of being on ART for a long time, which may lead to attrition from care [36].

Patients initiated ART regimens on d4T-based regimens as the preferred first-line ART regimen were less likely to experience mortality or LTFU at 12 months than those initiated on TDF-based first-line ART regimens, which is in contrast to results from previous studies [37–40]. The d4T based ART regimens were first-line drugs that were used early in the epidemic, in accordance with the HIV treatment guidelines from 2004 to 2010. As more evidence on safety and less toxic drugs became available, they were phased out of as part of preferred first-line ART regimen [39, 41, 42]. Though, becoming stable on treatment following exposure to these regimens would possibly lead to better treatment outcomes among these patients as they survived toxicities and advanced diseases and could adhere to treatment despite this adverse experience [43]. Perhaps these patients were also more like to achieve viral load suppression and had a reduced risk of comorbid conditions during the follow-up period, for the same reasons.

Weight gain after ART initiation is a sign of improving health status as HIV-infected patients initiate and remain on ART [44]. This has led to better patient outcomes in our cohort. However, being overweight or obese has recently become more prevalent among patients on ART, which may increase the risk of obesity-related diseases which are on the increase among ART patients [44–46].

We also found that Non-South African patients were less likely to be deceased or LTFU than South African patients. Which is similar to findings of a study by McCarthy K et al. (2009) that showed non-South African’s had better retention in care and lower mortality compared to South Africans [47]. Non-South Africans may be more motivated to remain in care because they may have had limited access to ART care in their home countries. Treatment access may be considered a great gain, contributing to greater retention but did not necessarily to greater adherence as their viral load suppression was similar to South Africans. GP down-referred patients had a higher portion of non-South Africans. Non-South Africans may prefer to be down-referred to GPs for improved access, there have been reports of maltreatment of foreign-nationals in public sector health facilities or denied access to health services if they do not have identity documents or resident permits [47, 48]. Therefore, GP down-referral may play a role in increasing ART access further for foreign nationals.

## Conclusions

As South African continues efforts to expand ART access to all HIV infected individuals, it is vital to expand efforts to support GPs to be able to manage public sector patients according to treatment guidelines to achieve good treatment outcomes. Our results show that GPs can contribute to caring for HIV patients on life-long ART following stabilization, but special efforts are required to improve retention in care.

### Limitations

The GP down-referral program required clinical stabilization as part of it is a selection criterion. Missing data in some key clinical data may bias outcomes. Misclassification as LTFU may be self-transfers to other health facilities or unreported mortality [35]. Results may not be generalizable to all settings as the referring site in a well-resourced tertiary hospital in an urban setting.

## Supplementary information

**Additional file 1.** Missing 12-month viral load.

## Data Availability

The datasets generated and/or analysed during the current study are not publicly available as the data are owned by the study sites and the National Department of Health (South Africa) and governed by the Human Research Ethics Committee (University of the Witwatersrand, Johannesburg, South Africa). All relevant data are included in the paper. The full data are available from the Health Economics and Epidemiology Research Office for researchers who meet the criteria for access to confidential data and with permission from the owners of the data. Contact the organization at information@heroza.org for additional information regarding data access.

## Abbreviations

3TC: Lamivudine
aHR: adjusted hazard ratio
aRR: adjusted relative risk
ART: antiretroviral therapy
AZT: zidovudine
BMI: body mass index
CCMT: comprehensive care management and treatment
CI: confidence interval
d4T: Stavudine
EFV: efavirenz
FTC: emtricitabine
GP: general practitioner
Hb: haemoglobin
HR: hazard ratio
IQR: interquartile range
LTFU: loss to follow-up
NHLS: National Health Laboratory Services
NVP: nevirapine
PHC: primary health care
PY: person-years
RR: relative risk
RtCHS: Right to Care Health Services
STI: sexually transmitted infection
TB: tuberculosis
TDF: tenofovir
UTT: universal test-and-treat
VL: viral load
