# Supplementary material for: Effective public-private partnerships for sustainable antiretroviral therapy: outcomes of the Right to Care health services GP down-referral program
Source: BMC Public Health. 2019 Nov 7;19:1471. doi: 10.1186/s12889-019-7660-x (PMC6836664; doi:10.1186/s12889-019-7660-x)
Supplement: Supplementary file 1 — Additional file 1. Missing 12-month viral load. [file 12889_2019_7660_MOESM1_ESM.docx]

**Additional file 1.** Missing 12-month viral load

|  | VL not missing | VL missing | Total | p values |
| --- | --- | --- | --- | --- |
|  | 2881 (78.2) | 804 (21.8) | 3685 (100.0) |  |
|  | n (%) | n (%) | n (%) |  |
| **Study cohort** |  |  |  |  |
| GP down-referred | 347 (93.0) | 80 (11.2) | 373 (100.0) | <0.001 |
| Clinic A | 1,901 (73.1) | 698 (26.9) | 2,599 (100.0) |  |
| Clinic B | 633 (88.8) | 26 (7.0) | 713 (100.0) |  |
| **Sex** |  |  |  |  |
| Female | 1,930 (79.2) | 508 (20.8) | 2,438 (100.0) | 0.04 |
| Male | 951 (76.3) | 296 (23.7) | 1,247(100.0) |  |
| **Age at down-referral/eligibility (years)** |  |  |  |  |
| 18-29.99 | 349 (81.0) | 82 (19.0) | 431 (100.0) | 0.23 |
| 30-39.99 | 1,178 (77.6) | 340 (22.4) | 1,518 (100.0) |  |
| 40-49.99 | 936 (77.2) | 276 (22.8) | 1,212 (100.0) |  |
| 50+ | 418 (79.8) | 106 (20.2) | 524 (100.0) |  |
| **Nationality** |  |  |  |  |
| South African | 2,562 (77.8) | 731 (22.2) | 3,293 (100.0) | 0.11 |
| Non-South African | 319 (81.4) | 73 (18.6) | 392 (100.0) |  |
| **Education level** |  |  |  |  |
| Primary school or less | 532 (80.6) | 128 (19.4) | 660 (100.0) | 0.17 |
| Some secondary school | 649 (79.3) | 169 (20.7) | 818 (100.0) |  |
| >=Grade 12 | 1,411 (82.0) | 309 (18.0) | 1,720 (100.0) |  |
| **Employment status** |  |  |  |  |
| Employed | 1,433 (76.4) | 444 (23.7) | 1,877 (100.0) | 0.007 |
| Unemployed | 1,383 (80.1) | 344 (19.9) | 1,727 (100.0) |  |
| **Time on ART at down-referral/eligibility (years)** |  |  |  |  |
| 1-2.99 year | 1,576 (84.5) | 289 (15.5) | 1,865 (100.0) | <0.001 |
| 3-4.99 years | 676 (73.1) | 257 (27.5) | 925 (100.0) |  |
| >=5 years | 629 (70.3) | 286 (31.1) | 895 (100.0) |  |
| **Guideline year of ART initiation** |  |  |  |  |
| 2004-2010 | 1778 (74.3) | 616 (25.7) | 2394 (100.0) | <0.001 |
| 2011-2014 | 1103 (85.4) | 188 (14.6) | 1291 (100.0) |  |
| **Baseline CD4 (cells/ul)** |  |  |  |  |
| 250-349.9 | 866 (84.3) | 161 (15.7) | 1,027 (100.0) | <0.001 |
| 350-499.9 | 951 (78.0) | 268 (22.0) | 1,219 (100.0) |  |
| >=500 | 1,064 (73.9) | 375 (26.1) | 1,439 (100.0) |  |
| **Baseline Haemoglobin (g/dL)** |  |  |  |  |
| **Baseline VL (copies/ml)** |  |  |  |  |
| <50 | 1,759 (78.6) | 479 (21.4) | 2,238 (100.0) | 0.67 |
| 50-199 | 711 (78.1) | 325 (22.5) | 910 (100.0) |  |
| 200-399 | 411 (76.8) | 124 (23.2) | 535 (100.0) |  |
| **Baseline BMI (kg/m2)** |  |  |  |  |
| Normal | 1,321 (86.6) | 204 (1.4) | 1,525(100.0) | 0.22 |
| Overweight | 729 (87.5) | 104 (12.5) | 833 (100.0) |  |
| Obese | 422 (84.9) | 75 (15.1) | 497 (100.0) |  |
| **ART regimen** |  |  |  |  |
| TDF+3TC/FTC+EFV/NVP | 1,069 (84.3) | 199 (15.7) | 1,268 (100.0) | <0.001 |
| AZT+3TC+EFV/NVP | 166 (85.6) | 28 (14.4) | 194 (100.0) |  |
| d4T+3TC+EFV/NVP | 1,619 (74.1) | 567 (25.9) | 2,186 (100.0) |  |
| **12 month Retention** |  |  |  |  |
| Alive and in care | 2,058 (71.4) | 376 (15.5) | 2,434 (100.0) | <0.001 |
| LTFU | 249 (83.0) | 51 (17.0) | 300 (100.0) |  |
| Deceased | 29 (87.9) | 4 (12.1) | 33 (100.0) |  |
| Transferred out | 545 (59.4) | 373 (40.6) | 918 (100.0) |  |

ART, antiretroviral therapy; VL, viral load; BMI, body mass index; 3TC, Lamivudine; ABC; EFV, efavirenz; TDF, tenofovir; NVP, Nevirapine; AZT, Zidovudine; FTC, emtricitabine; d4T, stavudine OR, odds ratio; 95% CI, 95% confidence interval
